# Exploring How Exposure to Truth and State-Sponsored Anti-Tobacco Media Campaigns Affect Smoking Disparities among Young Adults Using a National Longitudinal Dataset, 2002–2017

**DOI:** 10.3390/ijerph18157803

**Published:** 2021-07-23

**Authors:** David C. Colston, Yanmei Xie, James F. Thrasher, Sherry Emery, Megan E. Patrick, Andrea R. Titus, Michael R. Elliott, Nancy L. Fleischer

**Affiliations:** 1 Center for Social Epidemiology and Population Health, Department of Epidemiology, School of Public Health, University of Michigan, Ann Arbor, MI 48109, USA; xyanmei@umich.edu (Y.X.); nancyfl@umich.edu (N.L.F.); 2 Biostatistics Core, Rogel Cancer Center, University of Michigan, Ann Arbor, MI 48109, USA; 3 Department of Health Promotion, Education, and Behavior, Arnold School of Public Health, University of South Carolina, Columbia, SC 29208, USA; thrasher@mailbox.sc.edu; 4 Center for Population Health Research, Department of Tobacco Research, National Institute of Public Health, Cuernavaca 62100, Mexico; 5 Social Data Collaboratory, NORC at the University of Chicago, Chicago, IL 60637, USA; emery-sherry@norc.org; 6 Institute for Social Research, University of Michigan, Ann Arbor, MI 48104, USA; meganpat@umich.edu; 7 Department of Population Health, New York University Grossman School of Medicine, New York, NY 10016, USA; andrea.titus@nyulangone.org; 8 Department of Biostatistics, School of Public Health, University of Michigan, Ann Arbor, MI 48109, USA; mrelliot@umich.edu; 9 Survey Research Center, Institute for Social Research, University of Michigan, Ann Arbor, MI 48104, USA

**Keywords:** tobacco control, media campaign, Truth, health disparities, health equity, smoking

## Abstract

Background. Little is known regarding long-term impacts of anti-tobacco media campaigns on youth smoking and related disparities in the United States. Methods. We examined longitudinal cohort data from Monitoring the Future (MTF) between 2000 and 2017 in modified Poisson regression models to understand the long-term impacts of televised Truth and state-sponsored ad campaign exposure at baseline (age 18) on first cigarette and daily smoking initiation 1 to 2 years later (at modal ages 19/20). We also used additive interactions to test for potential effect modification between campaign exposure and smoking outcomes by sex, race/ethnicity, and parental educational attainment. Results. We found no evidence for baseline media campaign exposure to be associated with first cigarette or daily smoking initiation at modal age 19/20. Further, results showed no evidence for effect modification between campaign exposure and first cigarette or daily smoking initiation. Conclusions. We found no evidence that baseline Truth and state-sponsored ad exposure was associated with first cigarette or daily smoking initiation at follow up, nor did we find any evidence for effect modification by sex, race/ethnicity, or parental education. We hypothesize that anti-tobacco media campaigns might have had a short-term impact on smoking behaviors, though these effects were not sustained long term.

## 1. Introduction

Nearly 14% of individuals in the US ages 18 and older smoke cigarettes [1], and the vast majority report initiating their experimental and daily cigarette use prior to the age of 21 [2]. Because the majority of individuals who smoke start when they are young, state and federal governmental agencies, as well as nonprofit organizations such as the Truth Initiative, have launched anti-tobacco media campaigns focused on youth. Evaluations of these campaigns have shown that they are effective in reducing the likelihood of smoking or intent to smoke among youth [3,4,5,6,7,8]. However, most studies that use nationally representative data to examine the relationship between media campaigns and smoking behavior among youth have been cross-sectional, with little known about the long-term impacts of anti-tobacco media campaigns on tobacco use. The tobacco product and policy landscape has also shifted greatly in recent years, with youth increasingly using e-products [9,10], the continued rise in cigarette taxes [11], and the passage of Tobacco 21 and smoke-free laws [12,13], which could mean many of the existing media campaign evaluations are outdated.

Some research on anti-tobacco media campaigns has shown that exposure to advertisements could differ in efficacy across sociodemographic groups. For example, one study has shown that higher levels of Truth and state-sponsored campaign exposure has a stronger impact on reducing the likelihood of smoking among males than females [14]. Another study that analyzed state-sponsored media campaigns alone presented gender-stratified effect estimates that were not substantively different between men and women, but highlighted how campaign effects differed by race/ethnicity between groups of individuals that had different gender identities [7]. This finding raises the importance of understanding how multiple social identities can modify the impact of anti-tobacco media campaigns, and, hence, the need to evaluate campaign effectiveness for subgroups of the population. Regarding socioeconomic status (SES), one study found media campaigns were more effective in reducing the likelihood of smoking prevalence for 10th and 12th graders whose parents had lower educational attainment and 12th graders who definitely did not plan on attending college relative to their higher SES counterparts [14]. If media campaigns differ in their long-term effectiveness to improve smoking behaviors by sociodemographic status, they could have a considerable impact on tobacco-related health disparities, as individuals of lower SES are more adversely impacted by smoking across their lifespan than their higher SES counterparts, and racial/ethnic minority populations are less likely to successfully quit later in life [15].

Our study examines how Truth and state-sponsored anti-tobacco media campaigns impact youth smoking initiation using a national, longitudinal sample of young adults. Our study also analyzes whether media campaigns differed in effectiveness by sex, race/ethnicity, and socioeconomic status (SES).

## 2. Materials and Methods

### 2.1. Sample

We used data from the longitudinal arm of the Monitoring the Future (MTF) study [16]. Participants for the panel are drawn from the nationally representative samples of 12th graders each year, here including those from base year 2001–2015 with follow-up data one or two years later at modal age 19/20; follow-up data were collected in 2002–2017 [16,17]. Analytic sample sizes (detailed below) varied by outcome to capture the impact of anti-tobacco media campaign exposure among participants who had never smoked or respondents who had not smoked daily, as appropriate.

### 2.2. Smoking Outcome Variables

We investigated two outcomes at modal age 19/20: first smoking initiation and daily smoking initiation. Any smoking initiation since baseline was defined as having smoked cigarettes in the past 30 days at age 19/20, among individuals who had never smoked at baseline (*n* = 7502). Daily smoking initiation was defined as whether the participant smoked at least one cigarette per day in the past 30 days at age 19/20 vs. no smoking or nondaily smoking, among individuals who had never smoked or who were not daily smokers at baseline (*n* = 11,998).

### 2.3. Media Exposure

The independent variable was exposure to Truth and state-sponsored anti-tobacco media campaigns, which was measured using gross ratings points (GRPs), collected by Nielsen Media Research, for the top 75 designated market areas (DMAs) [18,19]. Our analyses used combined 12-month sums of GRPs, which were calculated by summing each respondent’s Truth and state campaign exposures and dividing by 100 to represent average views per person. The combined exposure variable pooled GRPs of Truth campaign from the American Legacy Foundation’s Truth campaign (2001–2008) and state-sponsored media campaigns from state health departments (2001–2015). We classified this combined exposure variable into quartiles.

### 2.4. Sociodemographic Variables

Individual-level sociodemographic variables included sex (male, female); race/ethnicity (non-Hispanic White, non-Hispanic Black, Hispanic/Latino, all other races not previously mentioned that do not identify as Hispanic/Latino—including those of multiple races); highest level of parental education (high school or less, some college, college or higher). While Hispanic or Latino individuals were categorized as one group for analyses, authors recognize there are important and distinct differences in geographic and cultural backgrounds [20], and conclusions should not necessarily be extrapolated to Hispanic or Latino populations, individually.

### 2.5. State-Level Covariates

To account for state-level characteristics that might impact the associations between media campaign exposure and smoking initiation, we included each state’s percent of Black individuals and percent of Hispanic individuals using data from the Survey of Epidemiology and End Results (SEER) population data [21]. We also included state percent living below the poverty line from the University of Kentucky’s Center for Poverty Research [22]. We controlled for the percentage of adults (age 25+) in each state with a bachelor’s degree or higher, using data from the United States Census Bureau (2000) and one-year estimates from the American Community Survey (2005–2015) [23,24]. Data were linearly interpolated between 2000 and 2005. To control for the taxation of tobacco products, we included an inflation-adjusted variable for the annual, state-level average cost of a cigarette pack from the CDC’s Tax Burden on Tobacco data [11]. Finally, we also included covariates to control for four census regions (Northeast, Midwest, South, and West) [25]. All covariates were based on each participant’s state at baseline.

### 2.6. Statistical Analysis

We conducted modified Poisson regression models to examine the relationship between media campaign exposure and the two smoking outcomes at modal age 19/20. All models control for baseline covariates: sex, race/ethnicity, parental education, census region, state-level cigarette price, state percent living below poverty, state percent of college graduates, state percent Black population, and state percent Hispanic population. We chose Poisson models with a sandwich variance estimator, rather than logistic models, in order to estimate relative risk [26].

Relative risks can be estimated by modified Poisson regression, which combines a log Poisson regression model with robust variance estimation. Assume *Y* is the smoking outcome of interest, *E* is the baseline media exposure, and *X* represents all baseline confounders including individual-level covariates and state-level covariates listed in Table 1. Then, the model can be written as

$$\log\left(P[Y=1|E,X]\right)=\beta_{0}+\beta_{1}E+\beta_{2}X$$

Therefore, the relative risks of the smoking outcomes in the three media exposure categories, 25–50 percentile, 50–75 percentile, and >75 percentile, versus the reference category (<25 percentile) are obtained by exp (*β*_1_).

We explored effect modification by sociodemographic variables by including a single interaction term between baseline media exposure and either sex, race/ethnicity, or parental education in separate models. We tested the significance of the interactions on the additive scale by calculating the average marginal effects (AME). To adjust for multiple testing, we applied the Benjamini–Hochberg correction with the false discovery rate at 5% across the interaction models for each outcome [27].

We applied attrition weights to account for attrition, oversampling of individuals who use drugs, and the complex survey design of the MTF study. Attrition weights were calculated as the inverse of the probability of participation at follow-up based on sex, race/ethnicity, college plans, truancy, high school grades, number of patents in the home, religiosity, parental education, alcohol use, cigarette use, marijuana use, other illicit drug use, region, cohort, and sampling weight.

Multiple imputation was performed to account for missing values using IVEware 0.3 [28]. Ten datasets were imputed under the missing at random assumption, with the exclusion of respondents who were missing attrition weights, did not respond, or have not yet aged into the first follow-up. The imputation model included cigarette use in last 30 days at baseline and at modal age 19/20, as well as all baseline covariates listed in Table 1, and other baseline characteristics: age, indicators for baseline year, school type, high school program, marital status, weekly earnings from a job, weekly earnings from allowances or other sources, ever smoked, 5 or more drinks in a row over the last 2 weeks, and marijuana use in last 30 days.

In sensitivity analyses, we investigated whether the inclusion of additional follow-up characteristics (attendance of a 4-year college, employment status, and full-time student status at modal age 19/20) would change the results. We also tested interactions between media exposure and the aforementioned follow-up covariates, which were additional markers of SES. We examined complete-case analyses for the main effects. We also tested whether there were differential effects of media campaigns on the outcome variables over time by the inclusion of an interaction term between media exposure and baseline year.

All analyses were conducted using Stata version 16.0 [29].

## 3. Results

Table 1 shows descriptive statistics for the analytic samples for any smoking initiation and daily smoking initiation at modal age 19/20. Media campaign exposure was distributed into approximate quartiles. For the any smoking initiation sample, 4.5% of respondents smoked at least one cigarette in the past 30 days at modal age 19/20. Daily smoking initiation was 4.9% at modal age 19/20 for the daily smoking initiation sample. The majority of respondents were female, non-Hispanic White, and had at least one parent with a college degree or more across samples.

The main associations of media campaigns on smoking outcomes at modal age 19/20 are reported in Table 2. Baseline media campaign exposure was not associated with any smoking initiation or daily smoking initiation at modal age 19/20.

Additive *p*-values from separate models including interactions between key sociodemographic characteristics and media campaigns are summarized in Table 3. We found no statistically significant interactions between media campaigns and sex, race/ethnicity, or parental education for any smoking initiation or daily smoking initiation at modal age 19/20.

In sensitivity analyses, we examined whether the inclusion of follow-up sociodemographic characteristics, including attendance of a 4-year college, employment status, and full-time student status at modal age 19/20, impacted the association between media campaigns and smoking outcomes. We found that the results were similar in magnitude and direction to the main results even with the addition of these variables (Table S1). We also tested effect modification by the follow-up characteristics and found no statistically significant interactions between media campaigns and any smoking initiation or daily smoking initiation by any of the characteristics (i.e., 4-year college attendance, employment status, or full-time student status) (Table S2). We also conducted complete case analyses for the main results and found that the results were consistent with those using multiple-imputed data, with one exception: individuals at >75th percentile of GRP had a higher risk of any smoking initiation than those at <25th percentile of GRP exposure (risk ratio (RR): 1.43, 95% CI: 1.01–2.03) (Table S3). Finally, we tested interactions between media campaign exposure and baseline year and found no evidence of differences in the relationships over time.

## 4. Discussion

Our longitudinal study of cohorts of 12th graders from a nationally representative sample found no evidence that greater exposure to anti-tobacco media campaigns at baseline was associated with any smoking or daily smoking initiation as youth transitioned into adulthood. Our findings are in contrast to the majority of previously published cross-sectional work, including a study analyzing cross-sectional MTF data from the same time period (2000–2015) [14], which found that higher levels of exposure to anti-tobacco media campaigns are generally associated with lower likelihoods of smoking intentions [3,6,7,14,30], smoking participation [5,6,7,14,31,32,33,34,35,36], and first cigarette initiation [14]. Only one study to date has analyzed the impact of campaign exposure on daily smoking initiation among youth and found no significant cross-sectional association between the two among 8th, 10th, and 12th graders [14].

Several other studies have utilized longitudinal cohort data to evaluate the impact of media campaigns on youth smoking behaviors, though they have relied primarily on self-reported exposure to anti-tobacco ads. Specifically, one study analyzing the Truth campaign between 2000 and 2002 found that self-reported recall of high, compared to low, anti-tobacco ad exposure over the past 12 months (defined as number of ads recalled), among students between 6th and 12th grade, was associated at follow-up with a lower likelihood of smoking intentions in the next five years, past 30-day smoking participation, and established smoking (having smoked 20+ days in past 30 days) [37]. A second study using MTF cohort data from 2001 to 2008 found that higher levels of anti-tobacco GRP exposure was not significantly associated with smoking uptake among 20- to 30-year-olds, which suggests the impact of anti-tobacco advertisement exposure on smoking initiation might not be sustained long term; however, the authors found that higher levels of ad exposure was associated with higher odds of quitting among all smokers and higher odds of quitting or reducing smoking among daily smokers, in a curvilinear fashion [38]. A third study used cross-sectional and longitudinal cohort data and found that high-sensation-seeking youth, ages 11 to 17, with confirmed awareness of concurrently running anti-tobacco ads in North Carolina in 2009 (cross-sectional arm) and in Fall of 2004 (longitudinal arm, compared to baseline data taken in Spring 2004), had lower odds of smoking experimentation and current smoking than those without confirmed awareness, though no differences were found for low-sensation-seeking youth [36]. A fourth study found that baseline awareness of anti-tobacco advertisements among Southern Californian 6th graders in 2000 was associated with a lower smoking susceptibility at 2- and 3-year follow-up [39]. These studies suggest a sustained effect of anti-tobacco campaigns on youth tobacco use when exposure is measured with self-report among adolescents. The one study that found no association used GRPs that reflect population-level exposure [40]. In contrast to self-reported exposure measures, GRPs do not capture potentially important individual-level variation in exposure within media markets [41]. Variability in youths’ media diets has grown as the media landscape has become increasingly fragmented [42,43]. Indeed, GRPs do not capture exposures online, where youth increasingly spend most of their media time [44] and where campaigns have targeted youth in more recent years [45]. Studies finding that media campaigns’ decreased effects took place before the recent acceleration of media fragmentation, and it is possible that GRPs have become less useful for evaluating campaigns that target youth. Finally, campaign effects may be most evident before the transition out of high school, which is the period covered in all studies that found effects. Both our and the only other study to find null effects examined that transition, when most youth change their living situations and become vulnerable to initiating a variety of risk behaviors. Campaigns that effectively address tobacco product use among young adults are sorely needed, as individuals between the ages of 18 and 24 have the highest prevalence of polytobacco use among any age group in the US [46].

We also found no evidence for effect modification between campaign exposure and either smoking outcome by sex, race/ethnicity, or parental education. These findings also differed from previous studies, although other studies have shown mixed results for different sociodemographic groups. For example, with respect to sex, one cross-sectional study utilizing MTF data between 2000 and 2015 showed that higher levels of Truth and state-sponsored media campaign exposure (GRPs) were more strongly associated with a lower probability of smoking participation among males than females [14], although another study using 1999–2003 cross-sectional MTF data that examined state-sponsored ad campaigns alone found no evidence for effect modification by sex [7]. Regarding race/ethnicity, the majority of studies support our finding that media campaigns do not differ in effectiveness by race/ethnicity [3,7,14]. With respect to SES, one study using 2000 to 2015 cross-sectional MTF data demonstrated that associations between Truth and state campaign exposure and smoking participation were stronger for individuals whose parents had lower educational attainment and who definitely did not plan to attend college when compared to their higher SES counterparts [14], which is consistent with previous work that individuals of lower SES react more strongly to anti-tobacco media campaigns compared to individuals of higher SES [47].

### 4.1. Significance

We hypothesize that, while Truth and state-sponsored ad campaigns likely have an effect on short-term smoking outcomes, as demonstrated by several cross-sectional and longitudinal studies, the effects might not be sustained over a longer period of time (e.g., at one and two years of follow-up, as measured by our study). Given the age of respondents in our sample, it is also possible that ads targeting youth are less effective in preventing smoking among 19- and 20-year-olds than for individuals in primary and secondary school. Further, young adult smoking rates have declined greatly in recent years, which could contribute to our lack of ability to find an association [48]. Finally, youth are shifting their attention from cable television to other streaming services and social media platforms [42], meaning televised ads could be less effective in recent years at reducing the likelihood of smoking behaviors among youth. This shift could also negatively impact tobacco control efforts, as tobacco companies such as Juul have launched campaigns, specifically related to e-cigarette use, on Twitter, Instagram, and YouTube [49]. Exposure to, and interaction with, online and social media-based pro-tobacco campaigns are associated with an increased likelihood of e-cigarette use [50], and the same could be true for cigarette smoking.

### 4.2. Limitations

This study was subject to several limitations. First, although the baseline MTF sample is a probability sample of US students in the 12th grade, the subsampling of the longitudinal sample does involve a degree of potentially non-random selection due to the need for contact information. Second, GRPs were measured at baseline, while smoking outcomes were measured at one- and two-year follow-up. As such, it is difficult to tease apart short and long-term effects of Truth and state-sponsored anti-tobacco campaigns, which appear to be different compared to studies that utilized cross-sectional and longitudinal data to evaluate concurrent or recently ended campaigns. As a result, respondents could be reporting awareness of an ongoing or recently completed campaign that is fresh in their memory, while our exogenous measure (GRPs) assessed exposure at baseline and did not factor in exposure to anti-tobacco media campaigns between baseline and follow-up. We attempted to limit this issue by examining the relationship only among respondents at the first follow-up time period. Additionally, while exogenous exposure measures such as GRPs can reduce the likelihood of recall bias relative to self-reporting, GRPs reflect an average exposure score but do not necessarily represent each respondent’s exposure, so it is possible that ads were not seen at the same intensity by a given individual that a GRP score might indicate, meaning that the true exposure might be overestimated. This is especially of concern given the fracturing media market among younger age groups [42,43,44]. Thus, self-reported measures may better account for individual-level exposure. Further, our media campaign data did not incorporate other forms of pro- or anti-tobacco media that occurred during this timeframe, including CDC-sponsored “Tips from Former Smokers” campaign ads, the Food and Drug Administration’s “Real Cost Campaign,” and the Truth “Finish It” campaign, which means we could be underestimating exposure to anti-tobacco media. Finally, we did not have access to target rating points (TRPs), which can more appropriately capture ad effects for specific age groups and might be particularly helpful when analyzing state-sponsored ads, which are broad and often targeted towards adults.

## 5. Conclusions

Our study found no evidence that exposure to televised Truth or state-sponsored anti-tobacco media campaigns in 12th grade was associated with any smoking or daily smoking initiation at modal age 19/20. Further, we found no evidence for effect modification between campaign exposure and either of our outcomes by sex, race/ethnicity, and socioeconomic status. Although exposure to anti-tobacco media campaigns appear to be effective in preventing youth smoking in the short term, these effects may not be sustained. Truth and state media campaign effectiveness may also be less reduced in recent years because youth are moving away from television and towards social media platforms, which were not evaluated in this study. More research is needed to understand how the impact of anti-tobacco ad exposure changes over time. Further, more should be done to understand the potential synergy of other ad campaigns and anti-tobacco ads on social media as youth transition to adulthood.

## Figures and Tables

**Table 1:** Weighted descriptive statistics for smoking initiation and daily smoking initiation analytic samples at modal age 19/20, monitoring the future longitudinal sample, baseline year 2001–2015. Results reflect imputed data (m = 10).

	Any Smoking Initiation	Daily Smoking Initiation
Past 30-Day Smoking		
Yes	4.5%	--
No	95.5%	--
Daily Smoking		
Yes	--	4.9%
No	--	95.1%
12 month non-depreciated		
≤25 percentile	27.5%	25.0%
25–50 percentile	24.3%	24.9%
50–75 percentile	23.4%	25.0%
>75 percentile	24.8%	25.1%
Sex		
Female	53.4%	52.7%
Male	46.6%	47.3%
Race/Ethnicity		
Non-Hispanic White	60.7%	61.6%
Non-Hispanic Black	14.0%	12.2%
Hispanic/Latino	16.8%	18.3%
Non-Hispanic Other	8.5%	7.9%
Education, Parents’ Highest		
≤High School	24.5%	26.2%
Some College	19.6%	20.6%
College +	55.9%	53.2%
Census Region		
Northeast	20.3%	20.0%
Midwest	22.9%	23.1%
South	32.6%	32.8%
West	24.2%	24.1%
Cigarette price (mean USD (SE), range)	5.9 (1.5), 3.8–10.6	5.8 (1.4), 3.8–10.6
% of state below poverty (mean % (SE), range)	13.3 (2.6), 5.4–22.2	13.3 (2.6), 5.4–22.2
% of state attained bachelor’s degree or higher (mean % (SE), range)	28.8 (4.6), 17.5–56.7	28.6 (4.5), 17.5–56.7
% of state identifying as Black (mean % (SE), range)	12.1 (6.7), 0.5–58.5	12.1 (6.8), 0.5–59.5
% of state identifying as Hispanic (mean % (SE), range)	17.1 (12.7), 1.7–48.2	17.0 (12.8), 1.1–48.2
N	7502	11,998

**Table 2:** Relative risk in media exposure (state + Truth) on any smoking initiation and daily initiation at modal age 19/20, monitoring the future longitudinal sample, baseline year 2001–2015. Results reflect imputed data (m = 10).

	Any Smoking Initiation		Daily Smoking Initiation	
	RR (95% CI)	*p* Value	RR (95% CI)	*p* Value
12-month non-depreciated (vs. <25 percentile)				
25–50 percentile	0.89 (0.56,1.41)	0.085	1.30 (0.95,1.78)	0.212
50–75 percentile	1.20 (0.78,1.84)		1.29 (0.94,1.77)	
>75 percentile	1.29 (0.86,1.92)		1.08 (0.78,1.51)	
N	7502		11,998	

**Table 3:** Unadjusted additive *p*-values associated with interaction terms between media campaign exposure and gender, race/ethnicity, and parental education for any smoking initiation, and daily smoking initiation at modal age 19/20, monitoring the future longitudinal sample, baseline year 2001–2015. Results reflect imputed data (m = 10).

	Any Smoking Initiation	Daily Smoking Initiation
Gender	0.635	0.490
Race/ethnicity	0.761	0.940
Parental education	0.280	0.288
N	7502	11,998

## Data Availability

Restricted Monitoring the Future Panel Data can be accessed at www.icpsr.umich.edu (accessed on 26 May 2021), though permission and approval are needed to access restricted data files.

## Supplementary Materials

The following are available online at https://www.mdpi.com/article/10.3390/ijerph18157803/s1, Table S1: Sensitivity analysis including follow-up characteristics. Relative risk in media exposure (state + Truth) on any smoking initiation and daily initiation at modal age 19/20, monitoring the future longitudinal sample, baseline year 2001–2015. Results shown are using imputed data (m = 10). Table S2: Additive *p*-values associated with interaction terms between media campaign exposure and follow-up characteristics for any smoking participation, and daily smoking initiation at modal age 19/20, monitoring the future longitudinal sample, baseline year 2001–2015. Results reflect imputed data (m = 10). Table S3: Relative risk in media exposure (state + Truth) on any smoking initiation and daily initiation at modal age 19/20, monitoring the future longitudinal sample, baseline year 2001–2015. Results shown are using complete cases.

## Abbreviations

RR	risk ratio
CI	confidence interval
