# Supplementary material for: Exploring How Exposure to Truth and State-Sponsored Anti-Tobacco Media Campaigns Affect Smoking Disparities among Young Adults Using a National Longitudinal Dataset, 2002–2017
Source: Int J Environ Res Public Health. 2021 Jul 23;18(15):7803. doi: 10.3390/ijerph18157803 (PMC8345400; doi:10.3390/ijerph18157803)
Supplement: Supplementary file 1 [file ijerph-18-07803-s001.zip › ijerph-1274668-supplementary.pdf]

Table S1. Sensitivity Analysis Including Follow-Up Characteristics. Relative Risk in Media Exposure (State+Truth) on Any Smoking Initiation and Daily Initiation at Modal Age 19/20, Monitoring the Future Longitudinal Sample, Baseline year 2001-2015. Results Reflect Imputed Data (m=10).

	**Any Smoking initiation**		**Daily smoking initiation**	
	RR (95% CI)	P value	RR (95% CI)	P value
12 month non-depreciated (vs. <25 percentile)				
25-50 percentile	0.83 (0.52,1.33)	0.087	1.29 (0.95,1.76)	0.201
50-75 percentile	1.08 (0.70,1.68)		1.31 (0.96,1.79)	
>75 percentile	1.23 (0.82,1.85)		1.09 (0.78,1.52)	
N	7138		11412	

Table S2. Additive P-values Associated with Interaction Terms between Media Campaign Exposure and Follow-Up Characteristics for Any Smoking Participation, and Daily Smoking Initiation at Modal Age 19/20, Monitoring the Future Longitudinal Sample, Baseline year 2001-2015. Results Reflect Imputed Data (m=10).

	**Any Smoking initiation**	**Daily smoking initiation**
Attended a 4-year college	0.911	0.348
Employment status	0.864	0.408
Full time student	0.870	0.488
N	7138	11412

Table S3. Relative Risk in Media Exposure (State+Truth) on Any Smoking Initiation and Daily Initiation at Modal Age 19/20, Monitoring the Future Longitudinal Sample, Baseline year 2001-2015. Results Reflect Complete Cases.

	**Any Smoking initiation**		**Daily smoking initiation**	
	RR (95% CI)	P value	RR (95% CI)	P value
12 month non-depreciated (vs. <25 percentile)				
25-50 percentile	1.00 (0.68,1.47)	**0.031**	1.21 (0.86,1.71)	0.253
50-75 percentile	1.41 (0.98,2.03)		1.28 (0.91,1.79)	
>75 percentile	**1.43 (1.01,2.03)**		1.00 (0.71,1.42)	
N	6686		10668	
